# 2.G. Workshop: The development of HTA collaboration in Europe: where we are and where we are going

**DOI:** 10.1093/eurpub/ckac129.084

**Published:** 2022-10-25

**Authors:** Organised by: EUPHA-HTA; EUPHAnxt, Chair persons: Chiara de Waure (EUPHA-HTA), Elena Petelos (EUPHA-HTA)

## Abstract

Health decision-making should be always well-informed. This draws the attention on the need to make the best use of available evidence in respect to different clinical and non-clinical issues to make a value-based allocation of resources. Health Technology Assessment (HTA) is a method for supporting evidence-informed health policymaking as it is defined as a multidisciplinary process that uses explicit methods to determine the value of a health technology at different points in its lifecycle. The role of HTA is of utmost importance at European Union level considering that citizens have the right to access healthcare in any EU country with reimbursement by their home country. Therefore, Member States (MS) cooperation in the evaluation of health technologies is envisaged to ensure safe, high-quality, and efficient healthcare. Several efforts have been put in place at EU level to make this collaboration possible. The cross-border healthcare directive 2011/24/EU have led to the establishment of a voluntary network of MS to facilitate their cooperation and the exchange of scientific information. This network has been supported by another network, namely the European Health Technology Assessment Network (EUnetHTA) that was launched in 2006 with the aim to increase the contribution of HTA to decision-making in EU MS, strengthen the link between HTA and health policymaking, and reduce the overlap and duplication of HTA efforts by promoting a more effective use of resources. EUnetHTA has created a long-lasting Joint Action that has developed principles, methodological guidelines, as well as functional online tools for the sharing production of HTA. In the meantime, starting from 2018, a proposal for an EU regulation on HTA has been discussed within the European Commission first and the European Council and Parliament later. The discussion led to the approval of the Regulation (EU) 2021/2282 on health technology assessment (HTAR) that entered into force on 11 January 2022 and will apply from 12 January 2025 onwards. The HTAR calls for mandatory participation in and use of joint clinical assessments at MS level opening a new era for HTA at EU level. In order to make public health professionals ready for the challenge, this skill-building seminar will provide the audience with basic knowledge in respect to the objective, the application, the methods, and the history of HTA at European level. After two formal presentations on these issues, a case study will lead the following interactive discussion with the audience.

**Key messages:**

• Health decisions should be informed by the determination of the value of health technologies through shared methods, such as Health Technology Assessment.

• Collaboration on Health Technology Assessment at European level has had a long-lasting story and is now looking out into a new challenging era that will be the EU regulation being implemented.

